# Patients’ experiences of humanising care in Scandinavian intensive care units - a systematic review

**DOI:** 10.1177/09697330261435046

**Published:** 2026-03-27

**Authors:** Monica Evelyn Kvande, Sanne Angel, Anne Højager Nielsen

**Affiliations:** 1 155319 Lovisenberg Diaconal University College, Oslo, Norway; 2 Research Unit of Nursing and Healthcare, Institute of Public Health, 1006 Aarhus University, Aarhus C, Denmark; 3 Department of Anaesthesiology and Intensive Care, Gødstrup Hospital, Herning, Denmark; 4 Department of Clinical Medicine, 1006 Aarhus University, Aarhus N, Denmark

**Keywords:** critical care, critical care nursing, humanism, intensive care units, systematic review

## Abstract

Using Scandinavian literature reporting patient experiences of intensive care as a case, the aim of this qualitative systematic review was to explore if humanising was addressed in the descriptions of patient experiences and, if so, what words and theoretical perspectives were used to underpin the results. A comprehensive search of qualitative studies published between 1 January 2016 and 12 December 2024 was conducted in Embase, CINAHL, MEDLINE, Scopus, and Web of Science. Twenty-nine studies reporting patient ICU experiences were included and analysed using thematic synthesis following Thomas and Harden. The review adhered to the Enhancing Transparency in Reporting the Synthesis of Qualitative Research checklist. The terms humanisation and de-humanisation were not explicitly used, but the studies engaged with these concepts by corroborating the three themes that defined humanising ICU in this study: (1) feeling recognised as a human being when experiencing connectedness with healthcare professionals, (2) feeling connected with themselves and the situation when experiencing safety and well-being, and (3) feeling connected with significant persons and life outside the ICU. In addition, our analysis identified a further theme: (4) experiencing capacity to influence the situation when able to express themselves. Humanising intensive care involves ethical and caring engagement with the patient, helping them feel connected, experience well-being, and influence their situation. Although Scandinavian literature has not directly studied humanisation, it has focused on understanding patients’ experiences and addressing the ontology of critical illness. As such, humanising ICU aligns well with Scandinavian nursing traditions.

**Implications for clinical practice:**

ICU staff should actively listen to patients and encourage them to express needs and preferences, create a safe and calm environment that supports well-being, and maintain communication with patients and families to promote a humanising approach to intensive care.

## Introduction

Advances in lifesaving technology and medical treatment have improved survival rates for critically ill patients admitted to intensive care units (ICUs) over the past decades.^1,2^ However, the human dimensions of care can be obscured by lifesaving technological and medical advances.^3^ A significant number of former ICU patients struggle to recover physically and psychologically and experience posttraumatic stress disorder, anxiety, and depression after hospital discharge.^4–6^ In response to patients’ suffering, there is an increasing interest in promoting a more humanised approach to intensive care,^7,8^ including a shift towards patient- and family-centred care that meets the individual beliefs and needs.^9^ Since 2014, the Spanish Proyecto HUCI (https://proyectohuci.com) has promoted a humanised approach to intensive care, focussing on areas such as open-door policies, communication, patient well-being, family involvement, end-of-life care, and humanised infrastructure.^8^ A scoping review^10^ revealed that humanising care was described as holistic care of the patient, the attitude of healthcare professionals toward patients, and humanisation as an organisational approach encompassing all subjects within the healthcare system. A systematic review by Nielsen, Kvande, and Angel^11^ synthesising findings from qualitative studies of patients’ ICU experiences found that intensive care was humanised when patients felt connected with healthcare professionals, with themselves and the situation when experiencing safety and well-being, and with significant persons and life outside the ICU.

In Scandinavia, healthcare is publicly funded, and ICUs are generally well staffed with nurse-patient ratios of 1:1 or 1:2, with no use of restraints. In this setting, the non-sedation paradigm that originated in early 2000^12,13^ has spurred an interest in patients’ experiences of intensive care.^14^ However, despite a long tradition for research in patients’ ICU experiences,^14^ the review by Nielsen et al.^11^ found only one Scandinavian study exploring patients’ experiences of humanising intensive care. This finding piqued our interest in whether the phenomenon of humanised ICUs might have been described using other terms in Scandinavian literature. If uncovered, such perspectives could provide valuable insights into how intensive care can be humanised to the benefit of ICU patients.

Therefore, using Scandinavian literature reporting patient experiences of intensive care as a case, the aim of this qualitative systematic review was to explore if humanising was addressed in the descriptions of patient experiences and, if so, what words and theoretical perspectives were used to underpin the results.

## Methods

### Design

This study employed a qualitative systematic review and thematic synthesis of qualitative studies following the method outlined by Thomas and Harden.^15^

### Inclusion criteria

Inclusion criteria include qualitative Scandinavian research papers exploring adult ICU patients’ experiences of intensive care. A previous scoping review^10^ mapped literature on humanising intensive care and identified the year 2016 as the inception point. We therefore limited our search from 1 January 2016 to 12 December 2024.

### Exclusion criteria

Exclusion criteria include the following: non-ICU setting, patients under 18 years, and end-of-life care; settings outside Denmark, Norway, and Sweden; reviews, conference abstracts, comments, editorials, and letters or not available in English, Danish, Swedish, or Norwegian.

### Selection of studies for review

**Figure 1.**
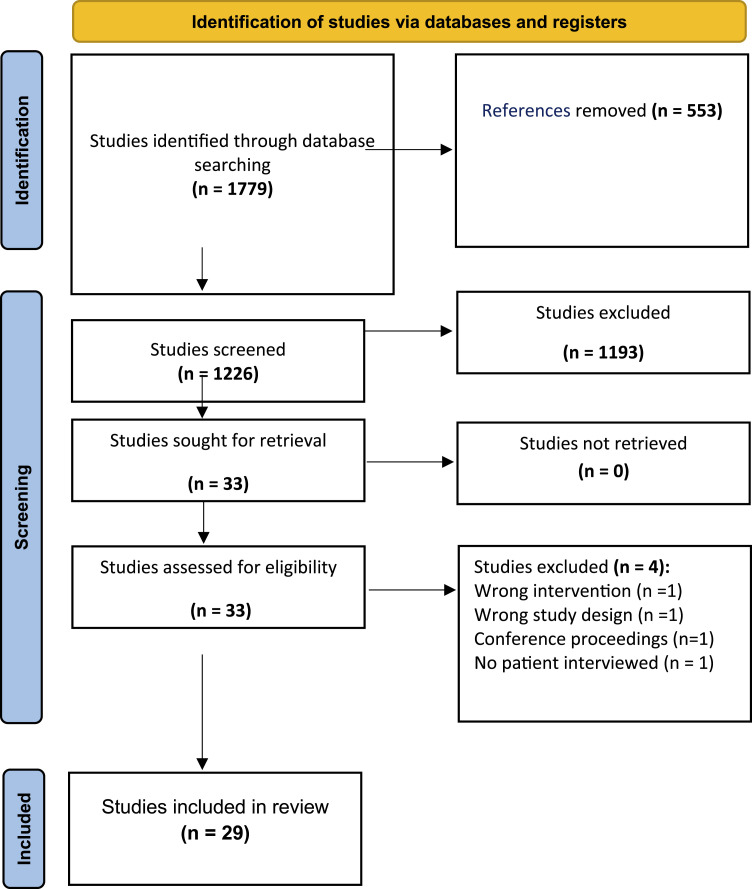
PRISMA diagram.

In April 2023, we searched Embase, CINAHL, MEDLINE, Scopus, and Web of Science for qualitative research papers reporting patients’ experiences of intensive care. In collaboration with the authors, a librarian experienced in research built a comprehensive and systematic search strategy in MEDLINE using medical subject headings and text words. The final search strategy in MEDLINE, shown in Appendix 1, was adapted for the other databases. The search was updated on 12 December 2024. The PRISMA flowchart (Figure 1) shows that a total of 1779 potentially relevant papers were identified. All identified studies were transferred to the reference management software Covidence, where 553 duplicates were removed. Two and two authors (MEK, SA, AHN) screened 1,226 papers based on title and abstract. In case of disagreement, the third author made the decision. Screening was done using the Covidence application, an internet-based application for handling, screening, and including papers in systematic reviews. A total of 29 papers were independently assessed for quality (Appendix 2) using the Critical Appraisal Skills Programme Qualitative Checklist (CASP-QC)^16^ by two authors and discussed until consensus was reached. 29 papers were included in the study.

### Data extraction

As we were exploring if humanising were addressed in descriptions of patients’ experiences of intensive care in a sample of Scandinavian literature that did not explicitly use the terms humanising or de-humanising, we organised the extraction and synthesis of data around two analytic questions: (1) How did patients’ experiences of intensive care reflect intensive care as humanising or de-humanising? and (2) What theoretical perspectives underpinned the results? The three themes from our previous study^11^ were used to guide our understanding of when intensive care could be experienced as humanising or de-humanising: (1) Patients felt recognised as human beings when they experienced connectedness with healthcare professionals; (2) experiencing safety and well-being helps patients feel connected with themselves and the situation; (3) feeling connected with significant persons and life outside the ICU.

The following data were extracted from the included papers: authors, publication year, journal, title, country of origin, objective, and research design/methodology. We extracted themes, quotes, and results pertaining to patients’ experiences of intensive care as humanising or de-humanising. Finally, the theoretical frameworks or studies used to discuss or underpin results pertaining to patient experiences of intensive care as humanising or dehumanising were extracted. Data extraction was handled in Microsoft Excel. All authors read all included papers, and extractions were discussed until agreement was reached.

### Synthesis of findings

Extracted excerpts from the included papers were read line by line and coded deductively using the three themes from our previous study as template^11^ and inductively, using open descriptive codes. The descriptive codes were subsequently used for organising the data into related areas and developing more comprehensive descriptions of each area into themes.

This was an iterative process of hermeneutic reading and writing, going back and forth between excerpts and the generated descriptive themes and a movement towards more interpretive themes that reached beyond the original papers^15^ and beyond the themes from our previous study.^11^ Interpretations were continually discussed by all authors until agreement was reached.

Descriptions of theoretical frameworks or studies were categorised and summarised, but no further interpretation was attempted. The study was reported using the Enhancing Transparency in Reporting the Synthesis of Qualitative Research (ENTREQ)^17^ (Appendix 3).

## Findings

A total of 29 papers (Table 1) met the inclusion criteria, each addressing patient experiences of intensive care in Scandinavia. Although the terms humanisation and de-humanisation were not explicitly used, the included studies engaged with these concepts by corroborating three previously described themes that were used to define humanising ICU in this study: Feeling recognised as a human being when experiencing connectedness with healthcare professionals, feeling connected with themselves and the situation when experiencing safety and well-being, and feeling connected with significant persons and life outside the ICU. Furthermore, our analysis extended this understanding by identifying an additional theme: Experiencing capacity to influence the situation when being able to express themselves (Table 2).

**Table 1.** Descriptive characteristics of the articles (*n* = 29).

Authors, year, country, and article number	Aim	Methodology, methods for data collection, and analysis	Participants	Findings	Theoretical framework for discussing findings
Alexandersen et al. (2019), Norwegian, 45	To explore aspects that promote or challenge long-term ICU patients’ inner strength and willpower	Hermeneutic‐phenomenological In‐depth interviews A hermeneutic‐phenomenological approach	17 patients	Two main themes and five subthemes: No doubt about coming back to life with subthemes: Strong connectedness to life, feeling alive and present, meaning and purpose and feeling valuable to somebody. How to ignite and maintain the spark of life with the subthemes: Practical solutions, coping skills from previous life experiences, provocative and inspiring experiences, and vivid dream experiences that ignite the willpower	Antonovsky A. (1996). «The salutogenic model as a theory to guide health promotion». Stroebe and Stroebe (1991) does ‘grief work’ work? Discussed in relation to other research literature
Berntzen et al. (2018), Norwegian, 44	To explore how critically ill patients treated according to a strategy of analgosedation experience and handle pain, other discomforts, and wakefulness	Exploratory, descriptive design Semi-structured interviews Systematic text condensation	18 patients, 10 patients re-interviewed	The main finding of the study was that patients treated with the analgosedation approach stated that physical pain was not a major concern. An overarching theme: ‘Pain relieved but still struggling’. Four main categories emerged from the analysis: ‘In discomfort, but rarely in pain’, ‘Struggling to get a grip on reality’, ‘Holding on’, and ‘handling emotionally trapped experiences’	Loeser and Melzack (1999) four broad pain categories and suffering as one category. Morse, J.M. (1992, 1999, and 2001) concept of comforting in nursing. Kolcaba K. (2003) description of comfort defines it as an outcome measure comprising relief, ease, and transcendence. Kean, S. et al., (2017) ‘ICU survivorship’ – A constructivists grounded theory of surviving critical illness Discussed in relation to other research literature.
Bjerregaard Alrø et al. (2024), Denmark, 29	To explore patients’ and relatives’ experiences of patients’ cognitive impairments while in the intensive care unit Ricoeur’s theory of interpretation	A multi-centre qualitative study, inspired by Ricoeur’s phenomenological-hermeneutic approach Participant observation and semi-structured single or dyadic interviews	20 patients (15 relatives)	Four themes: Having a hazy memory and a foggy brain Frustrations due to difficulties in speaking An altered sense of self, and feeling of disconnect between body and mind	Annachiara M. (2017). The ABCDEF bundle in critical care discussed in relation to other research literature
Bohart et al. (2023), Denmark, 18	To explore perspectives and wishes for patient- and family-centred care among adult patients and family members with recent experience of admission to an adult ICU	Qualitative, pragmatical Dyadic semi-structured interview Thematic analysis	8 ICU patients and 15 relatives	Ongoing dialogue is fundamental humanising Equipping family to navigate	Davidson JE, Aslakson RA, Long AC, et al. (2027). ‘Guidelines for family-centered care in the neonatal pediatric, and adult ICU’
Dreyer et al. (2024), Denmark, 43	To explore non-sedated patients experiences of live music in the ICU	Phenomenological-hermeneutic approach Observation and interviews Ricoeur’s theory of interpretation	18 patients	Five themes: A break from everyday life, a room with beautiful sounds and emotions, too tired to participate, knowing the music makes it meaningful, and a calm and beautiful moment	Discussed in relation to other research literature
Eklund et al. (2024), Sweden, 30	To examine patients’ experiences of receiving care in an ICU for COVID-19 and the subsequent rehabilitation process	Exploratory qualitative study Semi-structured interviews Thematic analysis	18 patients	Two themes: An isolated world with silver linings and recovery in the wake of the pandemic	Discussed in relation to other research literature
Flinterud et al. (2022) Norwegian 19	To explore and describe what intensive care patients experience as limiting and strengthening throughout their illness trajectories to reveal their needs for support and follow-up	Phenomenological approach Individual interviews Giorgi’s (2009) phenomenological analysis	10 patients	Being seen and met as individuals with unique personalities and traits was highlighted. Discussing everyday matters, their faith, or making jokes with the health care professionals looking after them were valued and viewed as essential when going through a difficult time	Galvin, K.M. and Todres, L. (2013). ‘*Caring and well-being: A lifeworld approach*’. Tembo, A.C. (2017) conceptual framework addressing existential suffering in intensive care, focussing on patients’ experiences of loss of control, isolation, and fear of death. Svenaeus, F. (2000 and 2011) health as homelikeness and illness as unhomelikeness being-in-the world discussed in relation to other research literature
Granberg-Axèll et al. (2020), Sweden 37	To investigate if there was a concordance between data from continuous clinical observations described in the researcher’s logbook and patients’ statements of their experiences of delirium during their ICU stay	A descriptive and qualitative multiple case–based design (Yin, R. K., 2009) Observation notes and notes from informal dialogues between the patient and observer. Information from patients’ medical records and nursing notes and brief interviews/reports from the patient nurse information from the ICU timetables; and interviews with patients following their discharge from the ICU Interpretative methods (Benner, P. 1994)	19 patients	There was a good concordance between clinical observations and patients’ statements of delirium. This indicates that early and subtle, as well as obvious, signs of delirium are possible to detect by attentive observations over time by analysing what patients are trying to convey by their speech, behaviour and body language	Morse, J.M. (1997) theory of suffering concept of comforting in nursing Eriksson K. (2006) theory of suffering human being. Discussed in relation to other research literature
Holm and Dreyer (2018), Denmark, 31	To explore the adult ICU patients’ experience of being conscious during endotracheal intubation and MV	Phenomenological-hermeneutic individual interviews Ricoeur’s theory of interpretation	4 patients	Three themes: The tube in the throat, to be conscious but feeling doped, and when passing of time is dragging on	Discussed in relation to other research literature
Holm and Dreyer (2017), Denmark, 36	To explore communication between adult, non-sedated, MV patients and nurses in the intensive care unit	Phenomenological hermeneutic approach Individual interviews Ricoeur’s theory of interpretation	9 patients	The main theme showed that communication is a movement between the two opposite feelings of comprehension and frustration. Sub-themes showed (1) the dynamics of power change when the patient is voiceless; (2) consciousness and voicelessness make caring difficult; and (3) the process of interpreting and structuring communication is situational	Discussed in relation to other research literature
Hylen et al. (2020), Sweden, 34	To explore the patients ´ experiences of pain when being cared for in the intensive care	An exploratory, qualitative design Individual interviews Thematic analysis	15 patients	Two main themes: (1) a lack of control with subthemes: Incapacitated – in body and in mind, unbalanced care, communication barriers and (2) to struggle for control with subthemes: Knowledge through communication, finding strategies and strength to handle the situation	Ekman I, Swedberg K, Taft C, et al. Person-centred care – ready for prime time. Marra A. E. et al., (2016 and 2017) ABCDEF bundle in critical care. Discussed in relation to other research literature
Kjeldsen et al. (2018), Denmark, 38	To explore patients’ experience of thirst while being conscious and mechanically ventilated	Hermeneutic approach Individual interviews Content analysis	12 patients	Four themes relating to the patients’ experiences of thirst during MV were identified: a paramount thirst, a different sense in the mouth, deprivation of the opportunity to quench thirst, and difficulties associated with thirst	Discussed in relation to other research literature
Køster et al. (2023), Denmark, 20	To provide an investigation of the experiences of isolation in COVID-19-positive patients in the ICU during the first phase of the COVID-19 pandemic in Denmark	Phenomenologically grounded qualitative research In-depth phenomenological interviews and observation studies Thematic analysis	6 patients	Themes consistently reported across all patients included (1) being objectified to degrees that implied self-alienation; (2) feeling a sense of being in captivity; (3) being in an experiential state of surrealism; and finally (4) experiencing extreme loneliness and intracorporeal deprivation	Discussed in relation to other research literature
Laerkner et al. (2017), Denmark, 35	To explore patients’ experiences of being awake during critical illness and mechanical ventilation in the intensive care unit	Interpretive description, an applied inductive, qualitative approach inspired by ethnography, grounded theory. and phenomenology Interviews and observation Qualitative, descriptive, and interpretive thematic analysis	20 patients in the first interview and 13 in the second interview. 17 patients participated in observations and interviews	A sense of agency and captured the patients’ ability to interact throughout the ICU stay. The familiar in the unfamiliar situation and captured initiatives experienced to facilitate familiar things and attitudes in an unfamiliar situation. Awareness of surrounding activities and describes the situated position of the awake, critically ill patient in the ICU surrounded by staff, technology, and other critically ill patients	Galvin, K.M. and Todres, L. (2013). ‘*Caring and well-being: A lifeworld approach*’. Jakimowicz S, Perry L. ‘A concept analysis of patient-centred nursing in the intensive care unit’. Discussed in relation to other research literature
Laerkner et al. (2019), Denmark, 39	To explore nurse-patient interactions in relation to the mobilisation of non-sedated and awake mechanically ventilated patients in the intensive care unit	Qualitative approach inspired by ethnography, grounded theory, and phenomenology Participant observation in the ICU, including interviews with nurses and patients Inductive, thematic analysis	13 patients (16 nurses)	Three themes: Diverging perspectives on mobilisation, and negotiation about mobilisation, inducing hope through mobilisation	Galvin, K.M. and Todres, L. (2013). ‘Caring and Well-being: a Lifeworld Approach’. Discussed in relation to other research literature
Lehmkuhl et al. (2024), Denmark, 46	To explore how patients with confirmed COVID-19 and their relatives experienced an intensive care unit stay and its significance for family dynamics	An exploratory qualitative study Dyadic interviews Inspired by Hochman’s dyadic analysis	19 patients (and their relatives)	Three themes: From ill to critically ill: The trauma of separation and fear of losing loved ones, the relatives’ significant role in creating a shared coherent understanding of the admission in ICU due to COVID-19, and the nurses’ roles as a go-between in maintenance of the family dynamic	Discussed in relation to other research literature
Lundberg et al. (2024), Sweden, 27	To explore experiences of care, psychosocial support, and well-being among COVID-19 patients 12–18 months after discharge	A qualitative approach with a descriptive design Semi-structured interviews on video-calls/telephone Qualitative content analysis	20 patients	Three categories: Being between life and death, i.e. relating to the participants state of being between life and death, the meaning of being seen and heard, i.e. relating to the value of being seen and heard, and connecting with normal life, i.e. relating to the striving for normalcy	Discussed in relation to other research literature
Nyhagen et al. (2023), Norway, 23	To explore communication between patients, family members, and nurses and to investigate previously unidentified communication challenges	A case-oriented design with multiple triangulations Participant observations and individual interviews An inductive, open approach at first followed by a more targeted investigation based on the theoretical framework created by Watzlawick et al. (1967)	9 patients (family members present at observation n = 12, clinicians present at observations n = 50). 6 interviews with family members, 7 with nurses, and 2 with physical therapist	Communication often seemed uncomplicated at the time of observations, but information from the interviews revealed another picture. Participants emphasised differently when they discussed their experiences, revealing a discrepancy in perceived importance in the situation. Family members had an important role in interpreting signs from the patient, uncovering challenges that would have been unknown to the nurses otherwise	Watzlawick P, Bavelas JB, Jackson DD. (1967). ‘Pragmatics of human communication: a study of interactional patterns, pathologies, and paradoxes’. Discussed in relation to other research literature
Nyhagen et al. (2024), Norway, 40	To describe different patterns of symptom communication in MV patients	Ethnographic Triangulation of participant observation and individual interviews Analysis was based on the principles of ethnographic research as described by Hammersley and Atkinson (2007)	9 patients (participant observation) and 6 patients (interviews) (50 clinicians: nurses, physiotherapists, and physicians)	Three patterns of communication: Proactive symptom communication, reactive symptom communication, and lack of symptom communication	Discussed in relation to other research literature
Olsen et al. (2017), Norway, 25	To investigate how adult patients experience their intensive care stay, their recovery period, and the usefulness of an information pamphlet	Qualitative exploratory Semi-structured in-person interviews Qualitative content analysis	29 patients	Main theme one was described as being on an unreal, strange journey remembering a mixture of fantasy and reality, which were linked to travelling in their sleep. Main theme two was described as normalising the abnormal, valuing family, and finding specific ways to cope with the strange journey. The second main theme was described as normalising the abnormal, valuing family, and finding unique ways to cope with the unfamiliar journey. Being on an unreal, strange journey and subthemes: Floating between facts and delusions and to understand and to be understood. Subtheme: 2b) valuing family	Discussed in relation to other research literature
Svenningsen et al. (2016), Denmark, 41	To describe the content of former intensive care unit patients’ memories of delusions	Phenomenological hermeneutical Telephone interview using the intensive Care Unit Memory Tool (CUMT) Analysis inspired by Ricour’s interpretive theory	114 ICU patients’	The ever-present family Dynamic spaces Surviving challenges constant motion	Discussed in relation to other research literature
Tengblad et al. (2024), Sweden, 42	To illuminate the experience of caring touch in intensive care from the perspectives of patients, next-of-kin, and HCPs	A caring perspective based on a lifeworld approach Qualitative observations and subsequent interviews with patients, next-of-kin, and HCPs Inductive content analysis	9 patients (4 next-of-kin, 8 nurses, and 8 assistant nurses)	One generic category: Caring touch creates presence with five subcategories: To touch and be touched with respect, touch as guidance and communication, touch causes suffering, touch creates compassion, and touch creates security	Discussed in relation to other research literature
Tingsvik et al. (2018), Sweden, 21	To explore the meaning of being a patient on mechanical ventilation during the weaning process in the intensive care unit	Hermeneutical phenomenological Semi-structured in-person interviews Hermeneutical phenomenological (Van Manen, 1997)	20 patients	Weaning was clearly intertwined with the lived experience of being a patient in the ICU and not a separate experience. The variations in the patients’ experiences of MV included the overall feeling of being a human being with a sense of stability and balance in life, to feelings of being in an unreal and strange environment	Ekman I, Swedberg K, Taft C, et al. «Person-centred care--ready for prime time». Eldh AC, Ekman I, and Ehnfors M. A comparison of the concept of patient participation and patients’ descriptions as related to healthcare definitions». Discussed in relation to other research literature
Vester et al. (2020), Denmark, 24	To explore the lived experience of being a couple during admission to an intensive care unit	A qualitative study grounded in a phenomenological-hermeneutic tradition Dyadic semi-structured interviews Ricoeur’s theory of interpretation	4. patients (4 spouse)	The life world of being a couple during admission to an intensive care unit was disclosed and divided into themes: For better and for worse, the meaningful proximity, and being a couple	Lögstrup KE. (1997) The Ethical Demand. Morse JM. Toward a praxis theory of suffering discussed in relation to other research literature
Vogel et al. (2021), Sweden, 26	To explore patients’ patterns of behaviour during the process from becoming critical ill to recovery at home	Grounded theory Interviews and observation Grounded theory (Glaser, 1998)	13 patients interviewed; 7 patients observed	The theory Stabilising life explains how patients’ main concern, being out of control, can be resolved. This theory involves two processes, recapturing life and recoding life, and one underlying strategy, emotional balancing that is used during the whole process	Kean, S. et al., (2017) ‘ICU survivorship’ - A constructivists grounded theory of surviving critical illness Kang, J. and Jeong, Y. J. (2018) A grounded theory approach on critical care survivors post-intensive care syndrome Discussed in relation to other research literature
Vogel et al. (2023), Sweden, 22	To explore memories and how they are experienced and manged by former patients who have been treated for COVID-19 in an intensive care unit	A qualitative descriptive design Interviews Thematic analysis	16 patients	Three themes: Distorted truth captive, and coping with memories	Lazarus and Folkman (1984) ‘Stress, Appraisal, and Coping’ discussed in relation to other research literature
Wallander Karlsen et al. (2019), Norway, 28	To explore the interaction between mechanically ventilated patients and healthcare personnel in intensive care units (ICUs), with a special emphasis on patients’ initiative to communicate	Phenomenological-hermeneutic approach Video-recorded observations Content analysis of observations	10 patients, 2 relatives, and 60 health care personnel	Four patterns of interaction were identified: immediately responded to, delayed response or understanding, intensified attempts, or giving up. Four domains of reasons for seeking attention: psychological expressions, physical expressions, social expressions, and medical treatment	Heidegger, M. (1996). Being and time: A translation of Sein und Zeit. Moore, C. (2014). Joint attention: Its origins and role in development. Discussed in relation to other research literature
Wallin et al. (2022), Sweden, 32	To describe how patients from the first wave perceived their COVID-19, their stay at an ICU, and remaining symptoms three to six months after intensive care	Prospective cohort study Questionnaires with open-ended questions Content analysis	64 patients	Memories from illness and hospital stay revealed in three categories: Awareness of the illness, a feeling of lost connection to reality, and being cared for in a dynamic environment	Discussed in relation to other research literature
Wermstrøm et al. (2017), Sweden, 33	To describe patients’ experiences of being cared for during invasive monitoring and treatment while awake	Exploratory qualitative design Semi-structured interviews Content analysis	8 patients (15 relatives)	Main theme: Sense of powerlessness and striving to regain control. Theme: To be exposed with subthemes: Loss of empowerment and to surrender Theme: To endure with subthemes: To feel hope, resting from the illness, and to participate	Gibson CH (1991) A concept analysis of empowerment. Discussed in relation to other research literature

**Table 2.** Example of coding process.

Author, publication, and year	Aim of study	Excerpt from study	Coding	Theme
Alexandersen et al. (2019)	To retrospectively explore the experiences of inner strength and willpower among long‐term ICU patients throughout their illness trajectory.	The pain from the treatment, e.g. an oral tube is experienced as negative. ‘Getting the sense of controlling a small piece in my life—that means a lot to me’.	Controlling even little aspects of the situation was valuable to the patient.	Experiencing capacity to influence the situation when being able to express themselves.
Berntzen et al. (2017)	To explore patients’ experience of pain, other discomforts, and wakefulness during critical illness when treated using the analgo-sedation approach.	When awake, patients felt in a position to contribute to decisions about their own treatment and care.	Just by being awake patients felt that they were in a position to influence their surroundings.	
Hylen et al. (2020)	To explore the patients ´ experiences of pain when being cared for in the intensive care.	Both verbal and non-verbal communication were used, such as grimacing, clenching one’s fists, and pointing at the body part that was hurting to communicate pain.	Being able to influence one’s situation by communicating pain through bodily symptoms that are understood by healthcare professionals. Thereby the patient could potentially achieve some kind of control over the situation.	
Holm et al. (2017)	To explore communication between adult, non-sedated, MV patients, and nurses in the ICU.	Comprehension occurred when nurses were able to interpret the patient’s message; when communication was unequivocal; when communication was about basic and/or informative subjects; and when there were continuity, patience, calmness, empathy, and time to prioritise communication.	Being understood by healthcare professionals can help patients overcome their feelings of being powerless.

**Table 3.** Theoretical references.

Theory	Reference	Article number
Theory on suffering	Eriksson^47^ Morse^48–50^	24 37 44
Theory on comfort	Kolcaba^51^	44
Theory on meaning	Antonovsky^52^	45
Theory on dwelling-mobility	Galvin and Todres^57^	19 35 39
Theory on coping	Lazarus and Folkman^55^	22
Theory on communication	Watzlawick et al.^56^	23
Theory on patient involvement, partnership, decision-making, and person-centred care	Davidson et al.^58^ Jakimowicz and Perry^59^ Ekman et al.^60^ Eldh et al.^61^	18 21 34 35
Philosophy on being	Heidegger^54^	28
Philosophy on ethical demand	Løgstrup^53^	24

All papers discussed their findings in the light of other studies or reviews. Four of twenty-nine papers discussed their reported aspects of humanising or dehumanising with reference to the literature on patient involvement, partnership, decision-making, and person-centred care. Seven papers referenced a metasynthesis by Egerod et al.^14^ about patient experiences of critical illness, thirteen papers referenced nursing theory and other philosophy, and only two referenced papers about humanising to discuss their findings.

### Feeling recognised as a human being when experiencing connectedness with healthcare professionals

Patients in the ICU expressed how important it was for healthcare professionals to respect them and treat them as valued human beings rather than seeing them as a critical illness or just a diagnosis.^18–22^ Healthcare professionals who showed interest in patients as individuals with unique personalities and everyday lives were important to patients’ experience of humanisation of care.^18–21,23,24^

Several studies described being looked in the eyes, smiled at, and touched with kindness as creating a connection between healthcare professionals and patients that humanised intensive care.^22,25–27^

‘The one most important thing was to have eye contact with the nurse’.^25^

Nurses who were attentive and patient during fearful moments of deterioration in the ICU managed to help patients feel more secure in their presence and provide human intensive care despite an uncontrollable medical state and environment.^19,21,24,28–32^ Diverting the patient’s thoughts from the difficult situation with laughter and jokes helped patients endure,^33^ which shows that establishing contact with patients is key to humanising care.

Patients’ interactions with nurses were not remembered as exclusively reassuring; some patients had memories of nurses being careful and cautious, and constantly focused on them during different caring actions, while other nurses acted more like machines,^22,25,29,30^ which again underscores the importance of the attitude and preparedness of healthcare professionals for connecting with patients.

Feelings of loneliness, alienation, isolation, and the loss of a sense of self could be countered by healthcare personnel when they established a relationship with the patient, thereby promoting humanised care.^20,30^ However, Køster et al.^20^ described the detrimental effects of not recognising patients as valued individuals.

‘Not only did patients experience being addressed as a “spreadsheet of numbers” and an “object of manipulation,” but this attitude towards the body was also internalized by the patients themselves’.^20^

This shows that objectification of patients influenced their sense of self negatively, whereas healthcare personnel’s efforts to acknowledge patients as valued persons may contribute to humanising intensive care.

#### Experiencing capacity to influence the situation when being able to express themselves

Patients emphasised that it was essential to be understood and were thankful when healthcare professionals managed to interpret their messages.^25,28,34–37^ However, patients expressed difficulties understanding the nurses and frustration being unable to express their basic human needs, such as thirst and the need for water.^25,38^ Being voiceless meant that patients could feel undignified or even humiliated when communication failed.^21,35,36,38^ Therefore, healthcare professionals’ willingness to communicate affected the degree to which patients felt in control,^28,29,33–36,39–41^ but involving patients in their care helped them to maintain self-esteem, made it possible to endure discomfort, and was perceived as a relief.

‘I was told not to touch, but they [nurses] discovered that I wouldn’t pull it [the endotracheal tube] out. Then they let me hold it and turn it a bit so that it didn’t irritate me in the throat’.^33^

Being allowed to be part of a decision regarding whether to start mobilisation or to wait and just do simple things like wiping their mouth or changing their body position was essential^21,35,39^ and a sign of being able to manage themselves.^33^ This shows that being conscious is key for patients to maintain a sense of influence, even if their participation is minimal.

#### Experiencing safety and well-being helps patients feel connected with themselves and the situation

Patients remembered both fantastic nurses and inexperienced, insecure nurses and emphasised that continuous care from experienced nurses was perceived as essential for feeling safe and secure in an unknown world.^25,27,35^ Feeling nurses’ presence and experiencing positive contact promoted a sense of safety and well-being.^19,32,42^

‘It was nice of them to try to make it a little humane; someone held my hand when they washed me’.^22^

Being cared for in a safe and calm environment also promoted well-being,^20^ whereas disturbing elements such as light or noise made rest impossible and promoted discomfort.^25^ Patients experienced feeling disconnected from their own body, changes in body functions, and feeling as though they were in a dream, floating between reality and delusions.^20,21,25,26,38^ Losing control over their body, feeling helpless, and becoming totally dependent on others affected the patients’ integrity and connection with themselves.^26,34,38^ Healthcare professionals’ initiatives to promote aesthetically founded well-being or the use of familiar aspects such as personal items in care contributed to the patients’ experience of feeling safe and secure in an unfamiliar world.^35,43^ This illustrates that healthcare professionals’ efforts to mitigate the strange and technological aspects of the ICU environment may help patients experience a sense of well-being and safety.

#### Feeling connected with significant persons and life outside the ICU

Patients remembered feeling safe and calm when family members were present. Even unconscious patients registered family members’ presence,^18,21,25,44^ which made them feel loved and cared for.^19^ Family members gave patients strength to carry on^24,45^ and motivated them to struggle.^21^ In addition, family members were experienced as a lifeline of hope for the patient and focused on the patient as a person and the patient’s familiar life, individual preferences, and situation.^19,21,23^

‘The adjacency of their close ones connected them to their familiar life, thereby creating a safe space in an unsafe existence’.^19^

Family members were described as the sole representatives of normality in the otherwise unfamiliar world of the ICU,^18,19^ which underscores the importance of supporting the bond with significant persons and life out of the hospital.^46^

#### Theoretical perspectives underpinning the results

In our search for the theoretical underpinnings of the articles’ findings on humanising aspects of intensive care, we looked at the discussion sections of the included studies. Mostly, the studies referred to other studies or reviews to support or refute their findings. Most prominent was a Nordic meta-synthesis of patients’ ICU experiences by Egerod et al.,^14^ in which being an ICU patient was aligned with a liminal state, where patients faced a choice between life and death before transitioning back to life.^20,21,28,35,41,44,45^

However, 13 studies also referred to theories or philosophy. Granberg-Axèll and Bergbom^37^ discussed their findings in the light of the Nordic nursing philosopher Professor Katie Eriksson’s work.^47^ In addition, we found discussions using Morse’s^48–50^ theory on suffering^24,44^ and Kolcaba’s^51^ theory on comfort.^44^

Some studies discussed their findings using philosophy or other theory. Alexanderson et al.^45^ referred to Antonovsky’s^52^ discussion of how patients make meaning in relation to inner strength and will to live, and Vester et al.^24^ used Løgstrup^53^ to support the importance of belonging to a couple and having mutual trust and obligation to care for one another with reference to ethical demand. Wallander Karlsen^28^ referred to Heidegger’s^54^ philosophy of being. The theory of coping^55^ was a reference in Vogel’s^22^ discussion, whereas Nyhagen^23^ referred to Watzlawick et al.’s^56^ theory on communication. The dwelling-mobility framework by Galvin and Todres^57^ was used to explore the tension between patients’ inability to act in their current situation and an orientation toward future possibilities.^19,35,39^

Finally, four studies^18,21,34,35^ discussed their findings in the light of patient involvement, partnership, decision-making, and person-centred care^58–61^ and only two recent studies referenced the literature on humanising intensive care (Table 3).^18,43^

## Discussion

The synthesis of data included for this qualitative systematic review confirmed the presence of humanising care defined as feeling recognised as a human being when experiencing connectedness with healthcare professionals, feeling connected with themselves and the situation when experiencing safety and well-being, and feeling connected with significant persons and life outside the ICU, and complemented this understanding by an additional theme: Experiencing capacity to influence the situation when being able to express themselves.

We searched for examples of humanisation of intensive care in a sample of Scandinavian literature where humanising and dehumanising care was not the focus of the studies and therefore not explored as such. Instead, the studies focused on different topics such as patient experiences of delirium,^37,41^ pain,^35^ inner strength and willpower,^45^ and communicating and being non-sedated during mechanical ventilation.^30^ Even if humanising has not been explicitly addressed in the Scandinavian literature, a great deal of attention has been paid to how nurses can mitigate patients’ experiences of being critically ill and cared for in the ICU,^18,20,31,34,36^ which is closely aligned with how humanising intensive care is described in other parts of the world such as holistic care of the patient, as an attitude of professionals toward patients, and as an organisational trait enabling humanised care for all subjects of the healthcare system.^8^ In this qualitative systematic review, attention to experiences in relation to intensive care may be influenced by the long practice of caring for non-sedated and non-restrained mechanically ventilated patients in the ICU which originated in Scandinavia around the turn of the millennium.^35,62^ Moreover, our review shows that caring for awake critically ill patients often calls for an understanding that the patients need human attention, comfort, kind touch, information, and communication, hence the concept of humanising was very much present, although not explicitly labelled as such.

The concept of humanising care including attention to patients’ human need for comfort, contact, and communication aligns well with the Nordic nursing tradition, which is rooted in an ethics of care, a philosophical and theoretical tradition influenced by the Norwegian nurse and philosopher Professor Kari Martinsen and Swedish/Finnish nurse and philosopher Professor Katie Eriksson. Martinsen and Eriksson have argued that care is and should be a prerequisite for good nursing.^63^ An ethics of care is reflected in our findings, which emphasise the contact between the healthcare provider and the patient as essential for feeling human and maintaining a sense of self. From this perspective, nursing that is attentive to all the patients’ expressed and unexpressed needs may be a necessary condition for humanising intensive care.

Our qualitative systematic review, however, did not only reveal positive patient experiences of care. There were also patient descriptions of alienation,^20^ isolation,^30^ lack of control,^33,34^ and inability to communicate.^29,36^ These negative experiences may lead to feelings of dehumanisation.^10^ Basile et al.^64^ explored what contributed to humanising and dehumanising care in the ICU and found that dehumanising care was insensitive care, callous talk, and lack of involvement. Similar to our findings, humanising care included connecting with the patient, encouraging and empathic communication, touching with care, respecting the patient as an individual, and inclusion of family members. Basile et al.^64^ underscored the importance of healthcare staff attitude and preparedness to engage with the patient in order to humanise intensive care. Rodriguez-Ruiz et al.^9^ argued that harmonising relationships between patients, healthcare professionals, and family members is key to establishing humanising care. This involves open communication, information sharing, and active listening which may be facilitated by patient and family-centred rounds. Notably, the authors also list a number of additional, but resource-demanding strategies to humanise the ICU including healing gardens and music therapy.^9^ While we do not disagree that such interventions may contribute positively to patient well-being, we consider ethical and caring engagement with the patient as the crux of humanising intensive care.

Our qualitative systematic review showed that most studies were discussed in light of other studies, and seven studies^20,21,28,35,41,44,45^ referenced the meta-synthesis of Nordic literature by Egerod et al.,^14^ which described the experience of intensive care as a liminal state between life and death, where the transition toward life is assisted by caring others. Egerod et al.^14^ described patients’ fight for survival as requiring caring relationships, with compassionate and competent nurses offering protection and providing strength and a connection to the real world. Their study aligns with the synthesis of international literature by Nielsen et al.,^11^ in which humanising was described as feeling connected to healthcare professionals, oneself, the situation, and family members. Therefore, the findings of this review and Egerod et al.^14^ indicate that parallel to the international humanising movement, Scandinavian literature has been preoccupied with the same problems, exploring patient lifeworld experiences from a mainly phenomenological and/or hermeneutical point of view.^14^ This focus suggests a strong interest in understanding the ontology of being critically ill and describing this experience in terms of caring, passionate, and protecting relationships with patients despite not using the term humanising.

Four papers^18,21,34,35^ discussed their findings in relation to patient involvement, partnership, decision-making, and person-centred care. Patient involvement^65^ and person-centred care^60^ are concepts that overlap with the concept of humanising intensive care.^8^ However, humanising intensive care transcends person-centred care as it is defined as both holistic care and a general attitude of professionals and healthcare systems toward patients, family members, and healthcare workers.^3,8^ Patient participation suggests involvement on professionals’ terms, while person-centred care is more holistic^60^; although both are inherently positive**.** Humanising care, however, is always accompanied by its negative counterpart – dehumanisation. In the ICU, patients’ humanity may be threatened by lifesaving technological and medical advances that improve survival rates.^3^ Findings from this review show that ICU nursing is concerned with mitigating patients’ negative experiences but does not perceive the ICU as dehumanising. The Spanish HUCI movement was sparked by a celebrity describing the ICU as a branch of hell.^66^ This suggests that the concept of dehumanising intensive care was originally a retrospective, out-side-in view of the ICU foreign to those who work there.^60,66^ This may explain why the term humanising intensive care is almost absent in Scandinavian literature on patient experiences of intensive care. Only two recent papers^18,43^ included references to humanising intensive care, which we believe reflects the growing global attention to the ICU humanisation movement in recent years.

## Limitations

This qualitative systematic review was built upon a search strategy ensuring that all literature studies were found and included. Furthermore, data extraction and analysis were undertaken by all three authors in collaboration, strengthening the internal validity of the study. The included sample allowed us to broaden our understanding of what humanising intensive care is. Literature was included from the year 2016, which marks the inception of the humanising ICU literature.^8^ The Scandinavian research tradition, however, is longer, and not including literature from before 2016 limits our ability to explore the fundaments of Scandinavian ICU patient experience research prior to year 2016. Thomas and Harden^15^ favour a representative sample over an exhaustive sample, and by including 29 very different studies, we have achieved that. We did not include studies from Finland, which is a limitation given that Finland and Scandinavia have comparable healthcare systems and a shared tradition of nursing theory.^67,68^

An advantage of thematic synthesis^15^ is that findings remain close to the data reported in primary studies, but the method has also been criticised for its lack of transparency.^69^ We have therefore provided examples of the coding process and supported all findings with examples from the studies included to provide transparency of the analytic process.

## Conclusion

This qualitative systematic review expands the understanding of humanising intensive care as an ethical and caring engagement with the patient which helps the patients feel connected, experiencing well-being and being able to influence their situation. Although the Scandinavian literature has not studied humanising per se, it has engaged with the idea by focussing on improving patients’ situations, influenced by a strong desire to understand the ontology of critical illness. In Scandinavia, humanising intensive care is rarely used as a concept; however, the ideas behind it are strongly present in Scandinavian literature and align well with the Scandinavian nursing tradition.

## Supplemental material


**Supplemental material -** **Humanising care in Scandinavian intensive care units – A systematic review**
Supplemental material for Humanising care in Scandinavian intensive care units – A systematic review by Monica Evelyn Kvande, Sanne Angel, and Anne Højager Nielsen in Nursing Ethics.
