# Supplementary material for: Patients’ experiences of humanising care in Scandinavian intensive care units - a systematic review
Source: Nurs Ethics. 2026 Mar 27;33(5):1479–503. doi: 10.1177/09697330261435046 (PMC13415904; doi:10.1177/09697330261435046)
Supplement: Supplemental material - Humanising care in Scandinavian intensive care units – A systematic review [file sj-pdf-1-nej-10.1177_09697330261435046.pdf]

Appendix 1 The complete search strings for the databases

We limited our search from 1 January 2016 to 12 December 2024. The search was updated on 12 December 2024

Database(s): **Ovid MEDLINE(R) ALL**
Search Strategy:

#	Searches	Results
1	Humanism/	
2	Holistic Nursing/	
3	Professional-Patient Relations/	
4	exp Patient-Centered Care/	
5	Patient participation/	
6	(humaniz* or humanis* or dehumaniz* or dehumanis* or holistic).ti,ab,kf.	
7	(centered* adj2 (patient* or person)).ti,ab,kf.	
8	(focus* adj2 (person* or patient*)).ti,ab,kf.	
9	(patient* adj3 (need* or experience* or satisf* or participat* or involv*)).ti,ab,kf.	
10	(communicat* or dignity or well-being or wellbeing or connected*).ti,ab,kf.	
11	(feel* adj2 safe*).ti,ab,kf.	
12	(support* adj2 (emotion* or psycholog* or patient*)).ti,ab,kf.	
13	(relation* adj2 (nurse* or professional*)).ti,ab,kf.	
14	(present adj2 centered*).ti,ab,kf.	
15	(care* adj2 presence).ti,ab,kf.	
16	(unique adj2 person*).ti,ab,kf.	
17	("life-world" or lifeworld).ti,ab,kf.	
18	(attention adj2 seek*).ti,ab,kf.	
19	or/1-18	
20	Critical Illness/	

21	Critical Care/	
22	exp Critical Care Nursing/	
23	Intensive Care Units/	
24	(icu or critical ill* or critically ill* or critical care or intensive care).ti,ab,kf.	
25	"Scandinavian and Nordic Countries"/	
26	(sweden or sverige or norway or norge or denmark or danmark or scandinavia* or nordic countries).mp.	
27	or/20-24	
28	25 or 26	
29	19 and 27 and 28	
30	limit 29 to yr="2024 -Current"	43

Scopus:

((TITLE-ABS-KEY ("Holistic Nursing")) OR (TITLE-ABS-KEY ((humaniz* OR humanis* OR dehumaniz* OR dehumanis* OR holistic))) OR ((TITLE-ABS-KEY (centered* W/2 patient*)) OR (TITLE-ABS-KEY (centered* W/2 person))) OR ((TITLE-ABS-KEY (focus W/2 person)) OR (TITLE-ABS-KEY (focus W/2 patient*))) OR ((TITLE-ABS-KEY (patient* W/3 need*)) OR (TITLE-ABS-KEY (patient* W/3 experience*)) OR (TITLE-ABS-KEY (patient* W/3 participat*)) OR (TITLE-ABS-KEY (patient* W/3 involv*))) OR (TITLE-ABS-KEY ((communicat* OR dignity OR well-being OR wellbeing OR connected*))) OR (TITLE-ABS-KEY (feel* W/2 safe*)) OR ((TITLE-ABS-KEY (support* W/2 emotion*)) OR (TITLE-ABS-KEY (support* W/2 patient*)) OR (TITLE-ABS-KEY (support* W/2 psycholog*))) OR ((TITLE-ABS-KEY (relation* W/2 nurse*)) OR (TITLE-ABS-KEY (relation* W/2 professional*))) OR (TITLE-ABS-KEY (present W/2 centered*)) OR (TITLE-ABS-KEY (care* W/2 presence)) OR (TITLE-ABS-KEY (unique W/2 person*)) OR (TITLE-ABS-KEY (("life-world" OR lifeworld))) OR (TITLE-ABS-KEY (attention W/2 seek*))) AND (TITLE-ABS-KEY (icu OR "critical ill*" OR "critically ill*" OR "critical care" OR "intensive care")) AND (TITLE-ABS-KEY (sweden OR sverige OR norway OR norge OR denmark OR danmark OR scandinavia* OR "nordic countries" OR finland OR greenland OR iceland)) AND PUBYEAR > 2024 AND PUBYEAR < 2025

58 hits

December 2024

Cinahl:

((((((MH "Humanism") OR (MH "Holistic Nursing") OR (MH "Professional-Patient Relations+") OR (MH "Patient Centered Care") OR (MH "Consumer Participation") OR (TI (humaniz* or humanis* or dehumaniz* or dehumanis* or holistic) OR AB (humaniz* or humanis* or dehumaniz* or dehumanis* or holistic)) OR (TI ((patient* or person) N2 centered*) OR AB ((patient* or person) N2 centered*)) OR (TI ((person* or patient*) N2 focus*) OR AB ((person* or patient*) N2 focus*)) OR (TI (patient* N3 (need* or experience* or satisf* or participat* or involv*) OR AB (patient* N3 (need* or experience* or satisf* or participat* or involv*)) OR (TI (communicat* or dignity or well-being or wellbeing or connected*) OR AB

(communicat* or dignity or well-being or wellbeing or connected*)) OR (TI feel* N2 safe* OR AB feel* N2 safe*) OR (TI (support* N2 (emotion* or psycholog* or patient*) OR AB (support* N2 (emotion* or psycholog* or patient*))) OR ((TI (relation* N2 (nurse* or professional*)) OR AB (relation* N2 (nurse* or professional*))) OR (TI present N2 centered* OR AB present N2 centered*) OR (TI care* N2 presence OR AB care* N2 presence) OR (TI unique N2 person* OR AB unique N2 person*) OR (TI ("life-world" or lifeworld) OR AB ("life-world" or lifeworld)) OR (TI attention N2 seek* OR AB attention N2 seek*))))))) AND (((((MH "Critical Illness") OR ((MH "Critical Care+") OR (MH "Critical Care Nurses+") OR (MH "Critical Care Nursing+")) OR (MH "Intensive Care Units") OR (TI (icu or critical ill* or critically ill* or critical care or intensive care) OR AB (icu or critical ill* or critically ill* or critical care or intensive care)))))) AND (((((MH "Scandinavia+") OR (TI (sweden or sverige or norway or norge or denmark or danmark or scandinavia* or nordic countries) OR AB (sweden or sverige or norway or norge or denmark or danmark or scandinavia* or nordic countries)))))

2023-2024

75 hits

Embase:

('humanism'/exp OR 'holistic nursing'/exp OR 'professional-patient relationship'/exp OR 'person centered care'/exp OR 'patient participation'/exp OR humaniz*:ab,kw,ti OR humanis*:ab,kw,ti OR dehumaniz*:ab,kw,ti OR dehumanis*:ab,kw,ti OR holistic:ab,kw,ti OR ((centered* NEAR/2 patient*):ab,kw,ti) OR ((centered* NEAR/2 person):ab,kw,ti) OR ((focus NEAR/2 person):ab,kw,ti) OR ((focus NEAR/2 patient*):ab,kw,ti) OR ((patient* NEAR/3 need*):ab,kw,ti) OR ((patient* NEAR/3 experience*):ab,kw,ti) OR ((patient* NEAR/3 satisf):ab,kw,ti) OR ((patient* NEAR/3 participat*):ab,kw,ti) OR ((patient* NEAR/3 involv*):ab,kw,ti) OR communicat*:ab,kw,ti OR dignity:ab,kw,ti OR 'well being':ab,kw,ti OR wellbeing:ab,kw,ti OR connected*:ab,kw,ti OR ((feel* NEAR/2 safe*):ab,kw,ti) OR ((support* NEAR/2 emotion*):ab,kw,ti) OR ((support* NEAR/2 patient*):ab,kw,ti) OR ((support* NEAR/2 psycholog*):ab,kw,ti) OR ((relation* NEAR/2 nurse*):ab,kw,ti) OR ((relation* NEAR/2 professional*):ab,kw,ti) OR ((present NEAR/2 centered*):ab,kw,ti) OR ((care* NEAR/2 presence):ab,kw,ti) OR ((unique NEAR/2 person*):ab,kw,ti) OR 'life-world':ab,kw,ti OR lifeworld:ab,kw,ti OR ((attention NEAR/2 seek*):ab,kw,ti)) AND ('intensive care'/exp OR 'critical illness'/exp OR 'intensive care nursing'/exp OR 'intensive care unit'/exp OR icu:ab,kw,ti OR 'critical ill*':ab,kw,ti OR 'critically ill*':ab,kw,ti OR 'critical care':ab,kw,ti OR 'intensive care':ab,kw,ti) AND (sweden:ab,kw,ti OR sverige:ab,kw,ti OR norway:ab,kw,ti OR norge:ab,kw,ti OR denmark:ab,kw,ti OR danmark:ab,kw,ti OR scandinavia*:ab,kw,ti OR 'nordic countries':ab,kw,ti OR finland:ab,kw,ti OR greenland:ab,kw,ti OR iceland:ab,kw,ti OR 'scandinavia'/exp) AND [2023-2025]/py**

152 hits

Appendix 2 Critical Appraisal Skills Programme Qualitative Checklist

Authors (year of publication)	Title	1. Was there a clear statement of the aims of the research?	2. Is a qualitative methodology appropriate?	3. Was the research design appropriate to address the aims of the research?	4. Was the recruitment strategy appropriate to the aims of the research?	5. Was the data collected in a way that addressed the research issue?	6. Has the relationship between researcher and participants been adequately considered?	7. Have ethical issues been taken into consideration?	8. Was the data analysis sufficiently rigorous?	9. Is there a clear statement of findings?	10. How valuable is the research?
Alexandersen et al.<br>2019	"Never in my mind to give up!" A qualitative study of long-term intensive care patients' inner strength and willpower-Promoting and challenging aspects.	+	+	+	+	+	+	+	+	+	Included
Berntzen et al.<br>2018	"Pain relieved, but still struggling"—critically ill patients experiences of pain and other discomforts during analgosedation	+	+	+	+	+	+	+	+	+	Included
Bjerregaard Alrø et al<br>2024	Cognitive impairment in intensive care unit patients: a qualitative exploration through observations and interviews	+	+	+	+	+	+	+	+	+	Included
Bohart et al. (2023)	Perspectives and wishes for patient and family centred care as expressed by	+	+	+	+	+	+	+	+	+	Included

	adult intensive care survivors and family-members: a qualitative interview study									
Dreyer et al. 2024	Live music in the intensive care unit - a beautiful experience	+	+	+	+	+	+	+	+	+	Included
Eklund et al 2024	Surviving COVID-19: patients' experiences of care and path to recovery	+	+	+	+	+	+	+	+	+	Included
Flinterud et al. 2022	Understanding the course of critical illness through a lifeworld approach.	+	+	+	+	+	+	+	+	+	Included
Granberg-Axèll et al. 2020	Intensive care unit delirium, clinical observations, and patients' statements: a case study.	+	+	+	+	+	+	+	+	+	Included
Holm & Dreyer 2015/2018	Nurse-patient communication within the context of non-sedated mechanical ventilation: a hermeneutic-phenomenological study	+	+	+	+	+	+	+	+	+	Included
Holm & Dreyer 2017	Intensive care unit patients' experience of being conscious during endotracheal intubation and mechanical ventilation	+	+	+	+	+	+	+	+	+	Included
Hylen et al. 2020	Patients´ experiences of pain in the intensive care - the delicate balance of control	+	+	+	+	+	+	+	+	+	Included

Kjeldsen et al<br><br>2018	Patients' experience of thirst while being conscious and mechanically ventilated in the intensive care unit	+	+	+	+	+	+	+	+	+	Included
Køster et al<br><br>2023	Experiences of isolation in patients in the intensive care unit during the COVID-19 pandemic	+	+	+	+	+	+	+	+	+	Included
Laerkner et al<br><br>2017	A sense of agency: an ethnographic exploration of being awake during mechanical ventilation in the intensive care unit	+	+	+	+	+	+	+	+	+	Included
Laerkner et al<br><br>2019	Negotiated mobilisation: an ethnographic exploration of nurse-patient interactions in an intensive care unit	+	++	+	+	+	+	+	+	+	Included
Lehmkuhl et al.<br><br>2024	Exploring critically ill patients' and their relatives' experiences of intensive care unit during COVID-19: a qualitative study	+	+	+	+	+	+	+	+	+	Included
Lundberg et al.<br><br>2024	Being between life and death—experiences of COVID-19 survivors 12 to 18 months after being treated in intensive care.	+	+	+	+	+	+	+	+	+	Included
Nyhagen et al<br><br>2023	Unidentified communication challenges in the	+	+	+	+	+	+	+	+	+	Included

	intensive care unit: a qualitative study using multiple triangulations.										
Nyhagen et al<br><br>2024	**Three patterns of symptom communication between patients and clinicians in the intensive care unit: A fieldwork study**	+	+	+	+	+	+	+	+	+	Included
Olsen et al.<br><br>2017	Evaluating the past to improve the future - a qualitative study of ICU patients' experiences.	+	+	+	+	+	+	+	+	+	Included
Svenningsen et al.<br><br>2016	Strange and scary memories of the intensive care unit: a qualitative, longitudinal study inspired by Ricoeur's interpretation theory	+	+	+	+	+	+	+	+	+	Included
Tengblad et al.<br><br>2024	"I am here"-the importance of caring touch in intensive care. A qualitative observation and interview study.	+	+	+	+	+	+	+	+	+	Included
Tingsvik et al.<br><br>2018	Patients' lived experience of intensive care when being on mechanical ventilation during the weaning	+	+	+	+	+	+	+	+	+	Included

	process: a hermeneutic phenomenological study.										
Vester et al. (2020)	The experience of being a couple during an intensive care unit admission.	+	+	+	+	+	+	+	+	+	Included
Vogel et al. 2021	Stabilizing life: a grounded theory of surviving critical illness.	+	+	+	+	+	+	+	+	+	Included
Vogel et al. 2023	The distorted memories of patients treated in the intensive care unit during the COVID-19 pandemic: a qualitative study	+	+	+	+	+	+	+	+	+	Included
Wallander Karlsen et al. 2019	Attention-seeking actions by patients on mechanical ventilation in intensive care units: a phenomenological-hermeneutical study	+	+	+	+	+	+	+	+	+	Included
Wallin et al. 2022	Intensive care-treated COVID-19 patients' perception of their illness and remaining symptoms	+	+	+	+	+	+	+	+	+	Included
Wermstrøm et al. 2017	From powerlessness to striving for control - experiences of invasive treatment while awake	+	+	+	+	+	+	+	+	+	Included

Appendix 3 Enhancing transparency in reporting the synthesis of qualitative research: ENTREQ Checklist

**Item No.**	**Guide and Description**	**Report Location**
1. Aim	State the research question the synthesis addresses	1, 4
2. Synthesis methodology	Identify the synthesis methodology or theoretical framework which underpins the synthesis, and describe the rationale for choice of methodology (e.g. meta-ethnography, thematic synthesis, critical interpretive synthesis, grounded theory synthesis, realist synthesis, meta-aggregation, meta-study, framework synthesis)	4
3. Approach to searching	Indicate whether the search was pre-planned (comprehensive search strategies to seek all available studies) or iterative (to seek all available concepts until they theoretical saturation is achieved)	4-5
4. Inclusion criteria	Specify the inclusion/exclusion criteria (e.g. in terms of population, language, year limits, type of publication, study type)	4
5. Data sources	Describe the information sources used (e.g. electronic databases (MEDLINE, EMBASE, CINAHL, psycINFO), grey literature databases (digital thesis, policy reports), relevant organisational websites, experts, information specialists, generic web searches (Google Scholar) hand searching, reference lists) and when the searches conducted; provide the rationale for using the data sources	4-5
6. Electronic Search strategy	Describe the literature search (e.g. provide electronic search strategies with population terms, clinical or health topic terms, experiential or social phenomena related terms, filters for qualitative research, and search limits)	4, 5 Appendix 1
7. Study screening methods	Describe the process of study screening and sifting (e.g. title, abstract and full text review, number of independent reviewers who screened studies)	5-6
8. Study characteristics	Present the characteristics of the included studies (e.g. year of publication, country, population, number of participants, data collection, methodology, analysis, research questions)	6-7 Table 1
9. Study selection results	Identify the number of studies screened and provide reasons for study exclusion (e.g. for comprehensive searching, provide numbers of studies screened and reasons for exclusion indicated in a figure/flowchart; for iterative searching describe reasons for study exclusion and inclusion based on modifications to the research question and/or contribution to theory development)	5. Figure 1

10. Rationale for appraisal	Describe the rationale and approach used to appraise the included studies or selected findings (e.g. assessment of conduct (validity and robustness), assessment of reporting (transparency), assessment of content and utility of the findings)	5, 6
11. Appraisal items	State the tools, frameworks and criteria used to appraise the studies or selected findings (e.g. Existing tools: CASP, QARI, COREQ, Mays and Pope [25]; reviewer developed tools; describe the domains assessed: research team, study design, data analysis and interpretations, reporting)	5. Appendix 2
12. Appraisal process	Indicate whether the appraisal was conducted independently by more than one reviewer and if consensus was required	5
13. Appraisal results	Present results of the quality assessment and indicate which articles, if any, were weighted/excluded based on the assessment and give the rationale	5. Appendix 2
14. Data extraction	Indicate which sections of the primary studies were analysed and how were the data extracted from the primary studies? (e.g. all text under the headings "results /conclusions" were extracted electronically and entered into a computer software)	5-6
15. Software	State the computer software used, if any	NA
16. Number of reviewers	Identify who was involved in coding and analysis	6
17. Coding	Describe the process for coding of data (e.g. line by line coding to search for concepts)	6
18. Study comparison	Describe how were comparisons made within and across studies (e.g. subsequent studies were coded into pre-existing concepts, and new concepts were created when deemed necessary)	6
19. Derivation of themes	Explain whether the process of deriving the themes or constructs was inductive or deductive	6
20. Quotations	Provide quotations from the primary studies to illustrate themes/constructs, and identify whether the quotations were participant quotations of the author's interpretation	6-11
21. Synthesis output	Present rich, compelling and useful results that go beyond a summary of the primary studies (e.g. new interpretation, models of evidence, conceptual models, analytical framework, development of a new theory or construct)	6-11
